# Hematocolpometra due to Imperforate Hymen

**DOI:** 10.1002/ccr3.71938

**Published:** 2026-01-28

**Authors:** Asae Inoue, Takuya Otsuki, Kosuke Ishizuka, Kasane Ikegami, Ruri Isono, Kenya Ie, Chiaki Okuse

**Affiliations:** ^1^ Department of General Internal Medicine St. Marianna University School of Medicine Kawasaki Kanagawa Japan; ^2^ Department of General Internal Medicine Kawasaki Municipal Tama Hospital Kawasaki Kanagawa Japan; ^3^ Department of General Medicine Yokohama City University School of Medicine Yokohama Kanagawa Japan; ^4^ Department of General Medicine Yokohama City University Medical Center Yokohama Kanagawa Japan; ^5^ Department of Pediatrics St. Marianna University School of Medicine Kawasaki Kanagawa Japan; ^6^ Department of Pediatrics Kawasaki Municipal Tama Hospital Kawasaki Kanagawa Japan

**Keywords:** hematocolpometra, hydronephrosis, imperforate hymen, prepubertal girl, urinary retention

## Abstract

Early recognition of imperforate hymen in premenarchal girls enables timely intervention to prevent serious complications; however, its nonspecific presentation often leads to missed diagnosis in primary care. Careful genital examination, supplemented by appropriate imaging, is essential when evaluating lower abdominal pain or constipation in girls with amenorrhea.

## Graphical abstract

2a: Significant fluid accumulation in the vaginal canal (yellow arrows) extending into the uterine cavity (red arrows), along with bladder distension (yellow arrowheads). 2b: Bilateral hydronephrosis and ureteral dilatation.

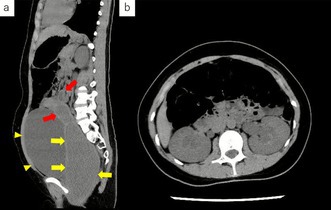

## 1 Case

**FIGURE 1:**
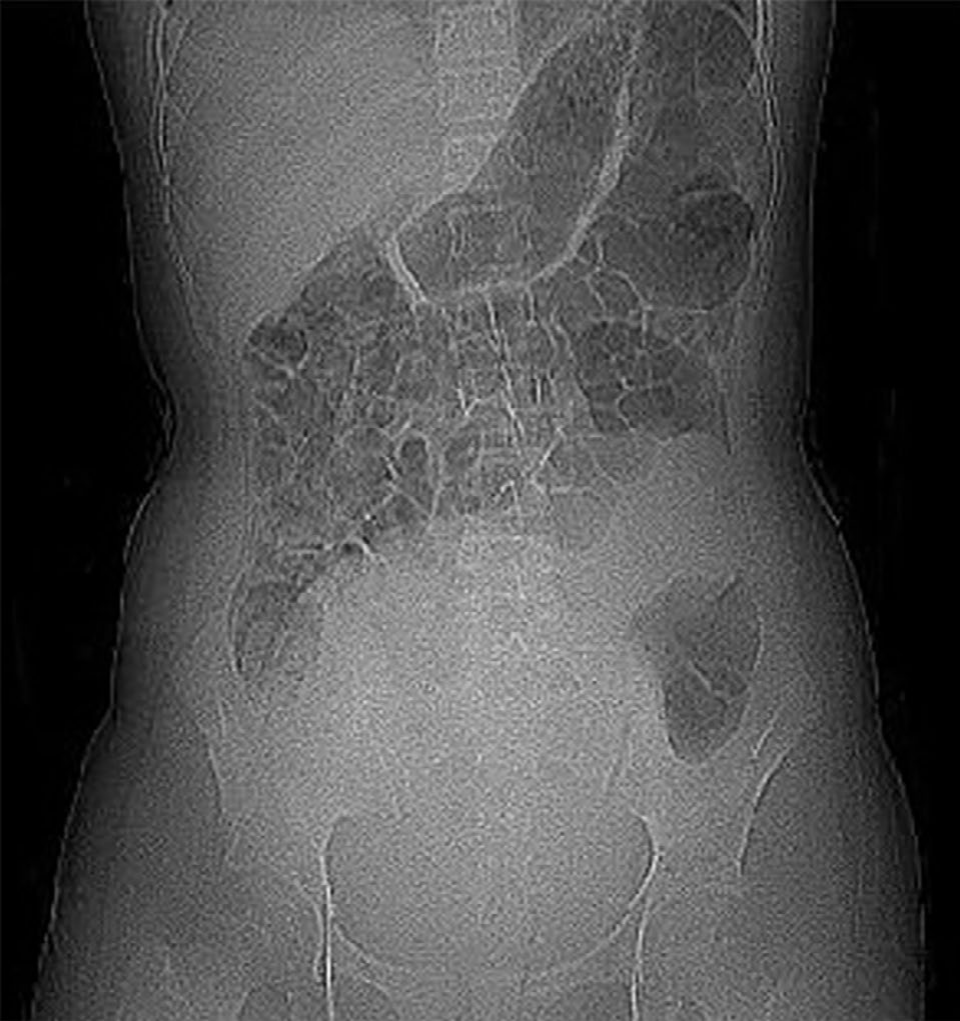
Abdominal X‐ray revealed decreased bowel gas and increased soft tissue density in the pelvic cavity, with cranial displacement of the gastrointestinal tract.

**FIGURE 2:**
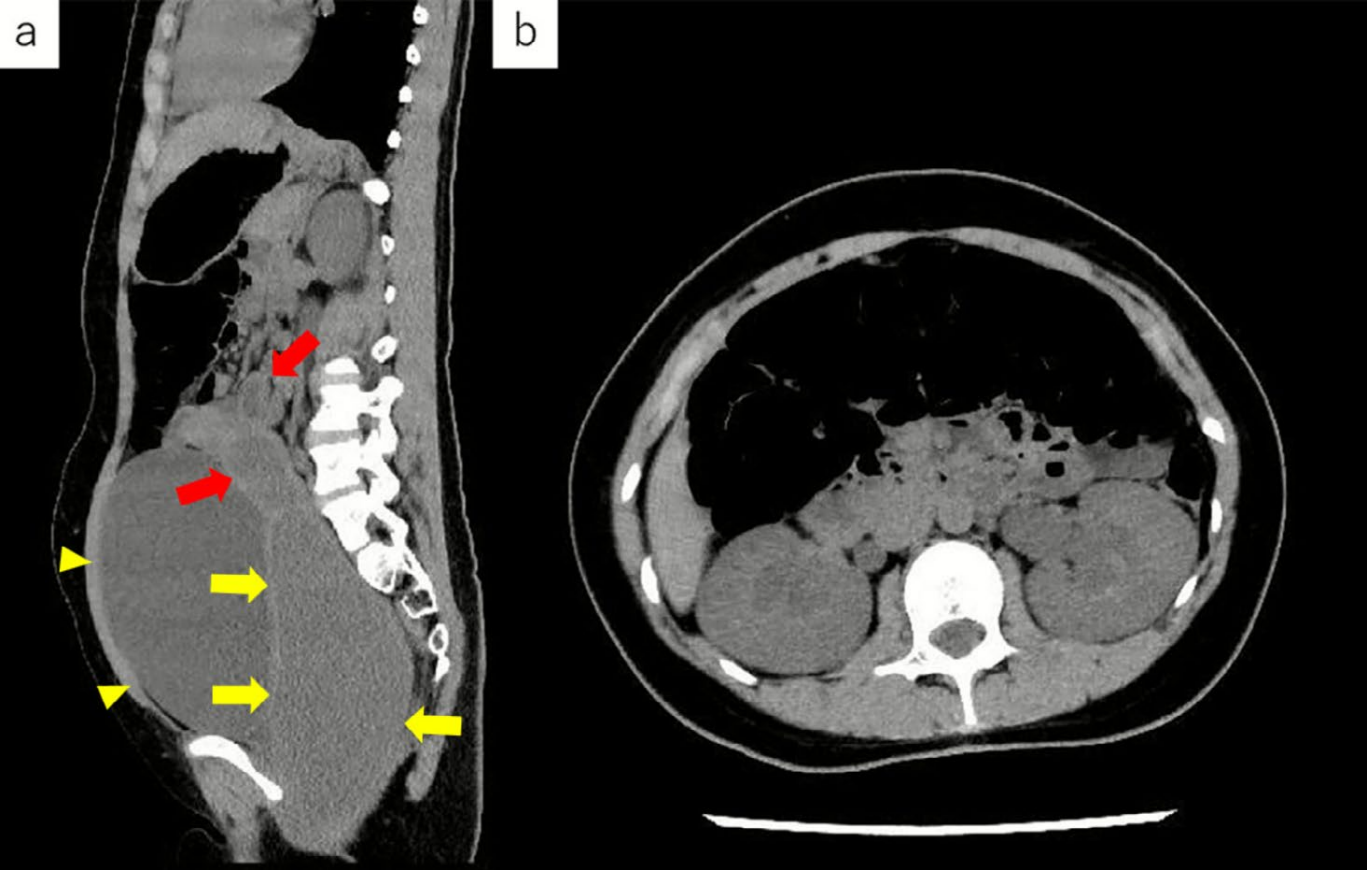
(2a) Sagittal CT demonstrated significant fluid accumulation in the vaginal canal (yellow arrows) extending into the uterine cavity (red arrows), along with pelvic organ prolapse and bladder distension (yellow arrowheads). (2b) Axial CT demonstrated bilateral hydronephrosis and ureteral dilatation.

A 9‐year‐10‐month‐old girl with a developmental disorder presented with a 4‐day history of lower abdominal pain and genital discomfort, accompanied by urinary and fecal urgency. She had not yet experienced menarche. Physical examination revealed marked abdominal distension, decreased bowel sounds, and diffuse lower abdominal tenderness. Genital examination showed an imperforate hymen and compression of the external urethral meatus. Abdominal X‐ray revealed increased soft tissue density in the pelvic cavity and cranial displacement of the bowel loops (Figure 1). Computed tomography showed significant fluid accumulation in the vaginal canal extending into the uterine cavity, pelvic organ prolapse, bladder distension, and bilateral hydronephrosis (Figure 2a,b). Based on these findings, the patient was diagnosed with hematocolpometra due to imperforate hymen, complicated by urethral compression and bilateral hydronephrosis. A hymenotomy was promptly performed, evacuating approximately 500 mL of bloody fluid, followed by vaginal irrigation, resulting in clinical improvement.

Imperforate hymen (IH) is an uncommon congenital anomaly of the urogenital sinus, with an incidence of 0.05%–0.1% [1], typically presents in early puberty with cyclic abdominal pain or voiding dysfunction. Owing to its low incidence and nonspecific symptoms, IH can be misdiagnosed as constipation or psychogenic disorders, leading to delayed diagnosis—especially when developmental disorders impede proper history‐taking, as seen in this case [2]. Without appropriate management, IH can cause infections, hydronephrosis, and endometriosis‐associated infertility [3]. Therefore, gynecological examination is essential in evaluating lower abdominal pain in premenarchal girls.

## Author Contributions

**Asae Inoue:** investigation, writing – original draft. **Takuya Otsuki:** writing – original draft, writing – review and editing. **Kosuke Ishizuka:** writing – review and editing. **Kasane Ikegami:** data curation, investigation, writing – review and editing. **Ruri Isono:** investigation, writing – review and editing. **Kenya Ie:** supervision, writing – review and editing. **Chiaki Okuse:** writing – review and editing.

## Funding

The authors have nothing to report.

## Consent

Written consent to use patient's case record (including X‐ray and Computed tomography) was obtained.

## Conflicts of Interest

The authors declare no conflicts of interest.

## Data Availability

Research data are not shared.
